# Identification and validation of stemness-related lncRNA prognostic signature for breast cancer

**DOI:** 10.1186/s12967-020-02497-4

**Published:** 2020-08-31

**Authors:** Xiaoying Li, Yang Li, Xinmiao Yu, Feng Jin

**Affiliations:** 1 Department of Breast Surgery, The First Affiliated Hospital of China Medical University, 155 Nanjing Road, Shenyang, 110001 China; 2 Department of Cell Biology, Key Laboratory of Cell Biology, Ministry of Public Health, and Key Laboratory of Medical Cell Biology, Ministry of Education, China Medical University, 77 Puhe Road, Shenyang, 110122 China

## Abstract

**Background:**

Long noncoding RNAs (lncRNAs) are emerging as crucial contributors to the development of breast cancer and are involved in the stemness regulation of breast cancer stem cells (BCSCs). LncRNAs are closely associated with the prognosis of breast cancer patients. It is critical to identify BCSC-related lncRNAs with prognostic value in breast cancer.

**Methods:**

A co-expression network of BCSC-related mRNAs-lncRNAs from The Cancer Genome Atlas (TCGA) was constructed. Univariate and multivariate Cox proportional hazards analyses were used to identify a stemness risk model with prognostic value. Kaplan–Meier analysis, univariate and multivariate Cox regression analyses and receiver operating characteristic (ROC) curve analysis were performed to validate the risk model. Principal component analysis (PCA) and Gene Set Enrichment Analysis (GSEA) functional annotation were conducted to analyze the risk model.

**Results:**

In this study, BCSC-related lncRNAs in breast cancer were identified. We evaluated the prognostic value of these BCSC-related lncRNAs and eventually obtained a prognostic risk model consisting of 12 BCSC-related lncRNAs (Z68871.1, LINC00578, AC097639.1, AP003119.3, AP001207.3, LINC00668, AL122010.1, AC245297.3, LINC01871, AP000851.2, AC022509.2 and SEMA3B-AS1). The risk model was further verified as a novel independent prognostic factor for breast cancer patients based on the calculated risk score. Moreover, based on the risk model, the low- risk and high-risk groups displayed different stemness statuses.

**Conclusions:**

These findings suggested that the 12 BCSC-related lncRNA signature might be a promising prognostic factor for breast cancer and can promote the management of BCSC-related therapy in clinical practice.

## Background

Breast cancer is the most commonly diagnosed malignancy and is the leading cause of cancer-associated mortality among women worldwide [1, 2]. In the field of clinical treatment, increasing attention has been focused on individual and precise therapeutic strategies. Thus, the identification of novel prognostic biomarkers and promising targets is considered to be an effective way to achieve this goal.

Heterogeneity is a hallmark of solid tumors, including breast cancer, which results from the enrichment of cancer stem cells (CSCs) [3, 4]. CSCs represent a dynamic subpopulation of tumor cells characterized by self-renewal, pluripotency and limitless proliferative properties [5]. Breast cancer stem cells (BCSCs) are considered the source of tumor aggression, metastasis, worse prognosis, chemoresistance and recurrence in breast cancer [6]. Therefore, identifying key stemness regulators of BCSCs is of great importance for both theoretical studies and clinical practice.

Long noncoding RNAs (lncRNAs) are a class of transcript RNAs longer than 200 nucleotides that are not translated into proteins [7]. LncRNAs are involved in the development and progression of various cancers at different levels, including epigenetic, transcriptional and posttranscriptional regulation, and are considered one of the most sensitive and specific cancer biomarkers [8–11]. Recently, lncRNAs have become a hot topic in stemness regulation of CSCs and prediction of prognosis in numerous cancers. Therefore, it is essential to identify key lncRNAs closely related to the stemness of BCSCs and prognosis in breast cancer.

In this study, we analyzed a dataset of lncRNA expression in breast cancers from The Cancer Genome Atlas (TCGA) and screened prognostic lncRNAs related to the stemness of BCSCs. We identified a 12 BCSC-related lncRNA signature with the ability to predict the survival prognosis of breast cancer patients.

## Methods

### Patient data sets

Clinical information and pathology records of patients with breast cancer were taken from the TCGA (https://cancergenome.nih.gov/). The edgeR package was used to normalize gene expression. A total of 1053 TCGA female breast cancer patients with lncRNA expression profiles were utilized in the present study.

Among them, 986 patients with complete follow-up information and survival time ≥ 30 days and 539 patients with complete clinicopathological data were applied to subsequent analyses. The clinical characteristics are detailed in Table 1.

**Table 1:** Clinical pathological parameters of patients with breast cancer

Feature	N (539)	%
Age (years)		
> 60	227	42.1
≤ 60	312	57.9
T classification		
T1 (< 2 cm)	147	27.3
T2 (2-5 cm)	323	59.9
T3 (≥ 5 cm)	55	10.2
T4 (chest wall and/or skin invasion)	14	2.6
N classification (pN)		
N0 (no metastasis)	259	48.1
N1 (1–3 metastasis)	178	33
N2 (4–9 metastasis)	64	11.9
N3 (≥ 10 metastasis)	38	7
M classification		
M0 (no distantmetastasis)	528	98
M1 (distant metastasis)	11	2
TNM stage		
I	96	17.8
II	318	59
III	114	21.2
IV	11	2
ER		
Negative	127	23.6
Positive	412	76.4
PR		
Negative	175	32.5
Positive	364	67.5
HER2		
Negative	440	81.6
Positive	99	18.4
Molecular subtypes		
HER2 amplification	92	17.1
Luminal A/B	419	77.7
TNBC	28	5.2

*T* tumor size, *N* lymph node, *M* distant metastasis, *TNM* *stage* according to AJCC 8th classification, *TNBC* triple-negative breast cancer

### Identification of BCSC-related lncRNAs in breast cancer

A total of 213 BCSC-related encoding genes (mRNAs) were extracted from the Molecular Signatures Database of Gene Set Enrichment Analysis (GSEA: M14079, M4740, M9246, and M13135). Finally, 1198 BCSC-related lncRNAs were identified by constructing BCSC-related mRNAs-lncRNAs co-expression network according to the criteria of |Correlation Coefficient|> 0.3 and p < 0.001 by Pearson correlation analysis using the limma R package [12].

### Identification of BCSC-related lncRNA prognostic signatures for breast cancer

To identify BCSC-related lncRNAs associated with survival, univariate Cox proportional hazards analysis was performed with p < 0.01 as the criteria, and multivariate Cox analysis was subsequently performed to establish the optimal prognostic risk model based on the Akaike information criterion (AIC = 1422.28) using the survival R package. Then, the risk score for each patient was calculated based on the following formula:

Risk score = coef (lncRNA1) × expr (lncRNA1) + coef (lncRNA2) × expr (lncRNA2) + …… + coef (lncRNAn) × expr (lncRNAn).

coef (lncRNAn) was defined as the coefficient of lncRNAs correlated with survival.

expr (lncRNAn) was defined as the expression of lncRNAs.

Breast cancer patients in the TCGA were divided into a high-risk group and a low-risk group according to the median risk score. Kaplan–Meier survival analysis was conducted to evaluate the survival difference between the two groups using the survival and survminer R packages.

### Independent prognostic analysis and ROC curve plotting

Univariate and multivariate Cox regression analyses were performed to assess the relationship between survival prognosis and age; estrogen receptor (ER) expression; progesterone receptor (PR) expression; human epidermal growth factor receptor 2 (HER2) expression; Tumor, Node, Metastasis (TNM) stage; tumor size (T); lymph node (N) metastasis; distant metastasis (M); and risk score using the survival R package. Time-dependent receiver operating characteristic (ROC) curves were plotted to evaluate the predictive accuracy for survival time through different clinical pathological factors and risk scores using the survival ROC R package.

### Statistical analysis

All statistical analyses were performed using R software (version 3.6.2). A co-expression network of BCSC-related lncRNAs-mRNAs with prognostic value in breast cancer was constructed visualized by Cytoscape and Sankey diagram. The correlation between the expression of BCSC-related lncRNAs and clinicopathological factors was analyzed by ggpubr R package. Principal component analysis (PCA) was performed for effective dimension reduction, pattern recognition, and exploratory visualization of high-dimensional data of the whole genome, 213 BCSC-related encoding genes and the risk model of BCSC-related lncRNAs expression profiles, respectively [13, 14]. GSEA was used for functional annotation. Two-tailed p < 0.05 was considered statistically significant.

## Results

### Identification of significant prognostic value of BCSC-related lncRNAs in breast cancer

**Fig. 1:**
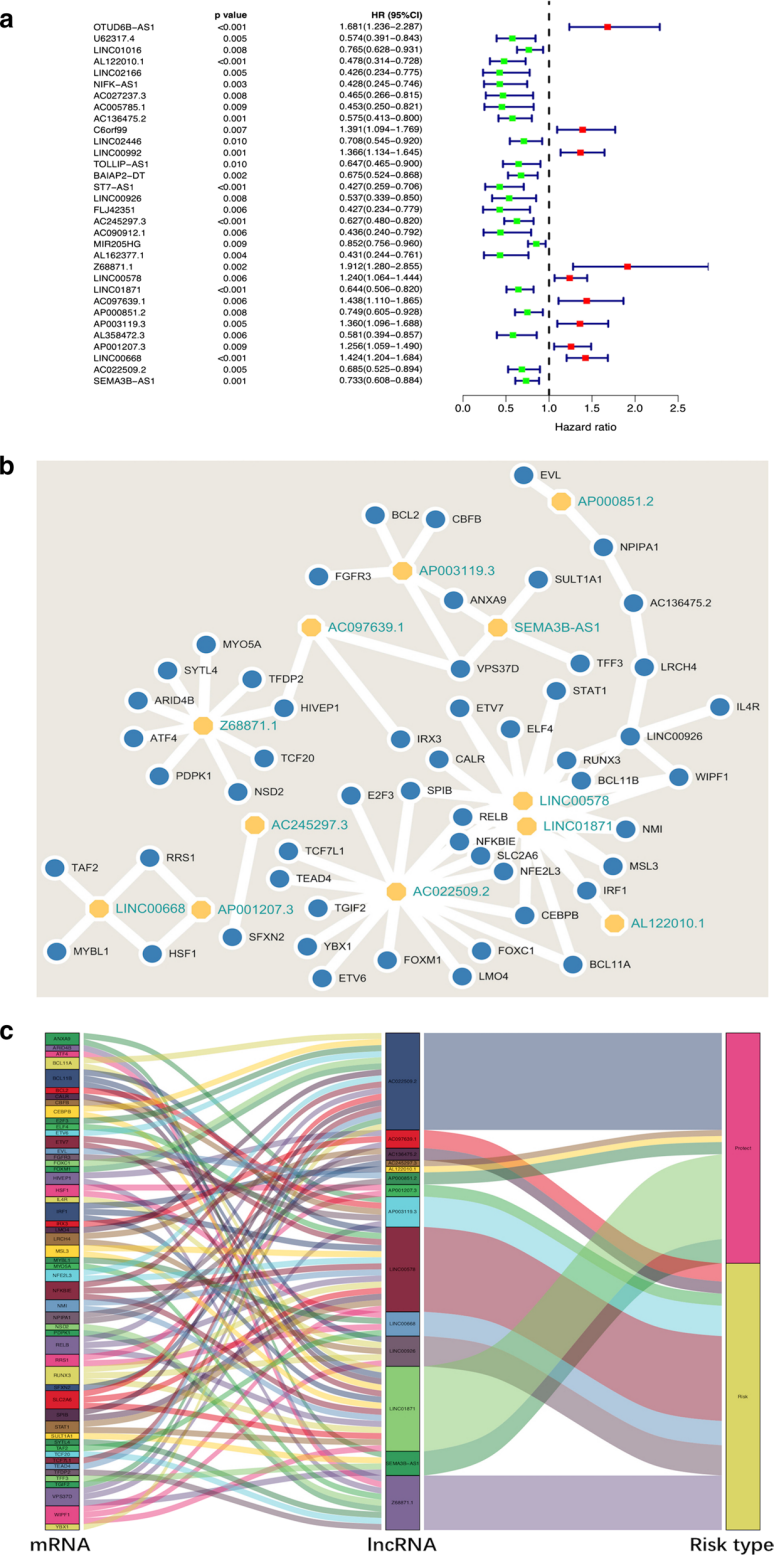
Identification of BCSC-related lncRNAs with significant prognostic value in breast cancer. **a** The forest showed the HR (95%CI) and p-value of selected lncRNAs by univariate Cox proportional-hazards analysis. **b**, **c** A co-expression network of the 12 BCSC-related lncRNAs-mRNAs with prognostic value was constructed and visualized using Cytoscape and Sankey diagram

**Fig. 2:**
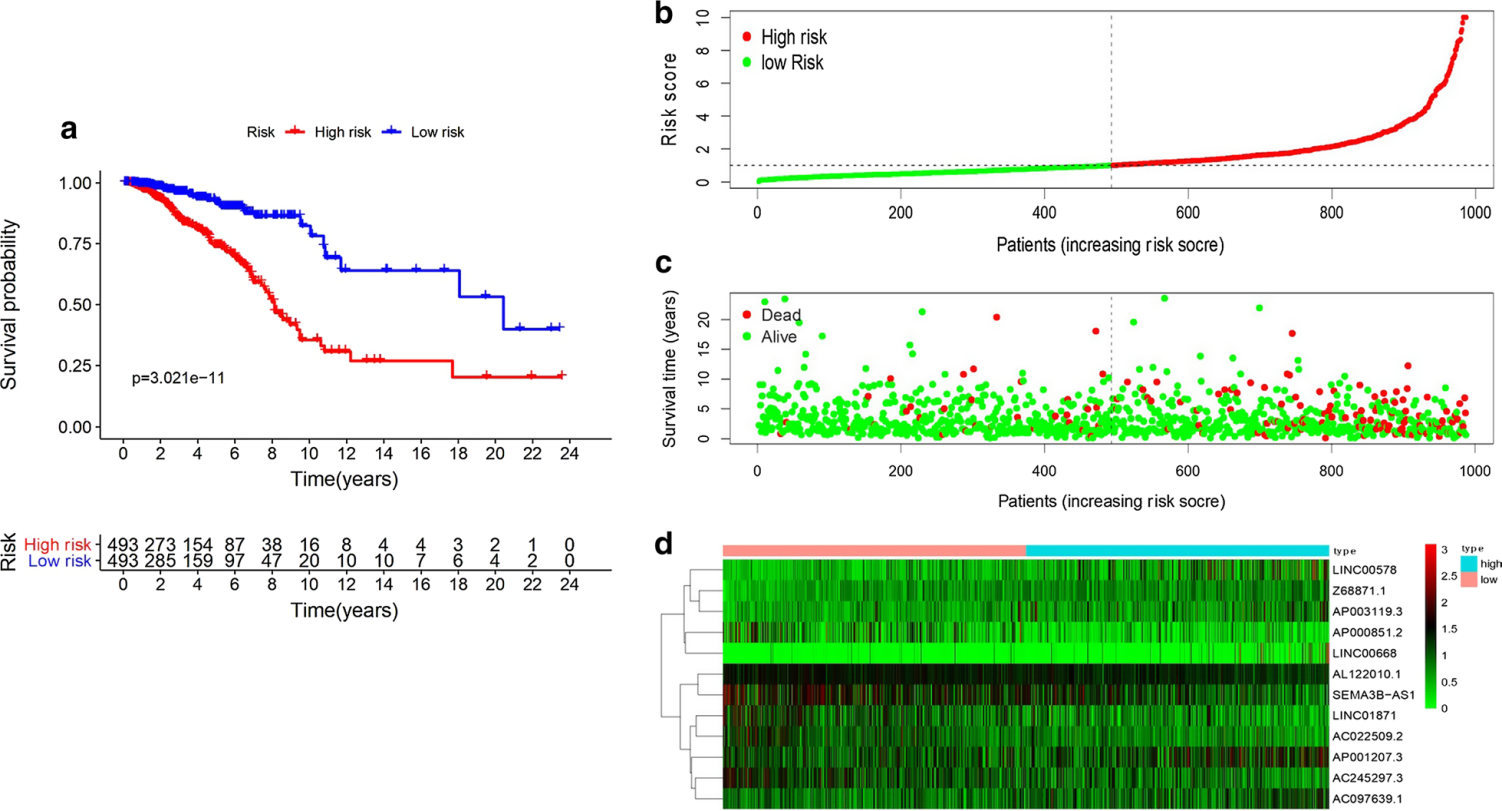
The prognostic value of the risk model of the 12 BCSC-related lncRNAs in the TCGA cohort. **a** Kaplan–Meier survival analysis of the high-risk and low-risk groups based on the risk model and median risk score. **b** The risk curve based on the risk score of each sample. **c** The scatterplot based on the survival status of each sample. The green and red dots represent survival and death, respectively. **d** The heatmap displayed the expression levels of BCSC-related lncRNAs in the high-risk and low-risk groups

A total of 1198 BCSC-related lncRNAs were obtained by constructing co-expression networks with 213 BCSC-related encoding genes (mRNAs) (Additional files 1, 2 and 3). Among them, 32 BCSC-related lncRNAs were significantly associated with the survival of breast cancer patients from the TCGA (p < 0.01) by Cox proportional hazards analysis, including 23 lncRNAs with low risk (hazard ratio (HR) < 1) and 9 lncRNAs with high risk (HR > 1) (Fig. 1). Subsequently, multivariate Cox analysis further screened 12 lncRNAs from the above 23 BCSC-related lncRNAs with prognostic significance, namely, Z68871.1, LINC00578, AC097639.1, AP003119.3, AP001207.3, LINC00668, AL122010.1,

AC245297.3, LINC01871, AP000851.2, AC022509.2 and SEMA3B-AS1 (Table 2). These 12 lncRNAs were constructed into the optimal prognostic risk model of BCSC-related lncRNAs. As shown in Fig. 1a–c visualization co-expression network of BCSC-related lncRNAs-mRNAs with prognostic value was established. According to the risk score formula and the calculated median risk score, breast cancer patients were divided into a high-risk group and a low-risk group. Kaplan–Meier survival analysis showed that the high-risk group presented worse overall survival (OS) than the low risk group (p = 3.021E−11) (Fig. 2a), suggesting that the risk score had prognostic value. The risk curve and scatterplot were made to illustrate the risk score and the corresponding survival status of breast cancer patients. The results showed that the higher the risk score was, the more mortality occurred (Fig. 2b, c). The heatmap of the expression of these 12 BCSC-related lncRNAs in breast cancer samples showed that Z68871.1, LINC00578, AC097639.1, AP003119.3, AP001207.3 and LINC00668 were upregulated in the high risk group, while AL122010.1, AC245297.3, LINC01871, AP000851.2, AC022509.2 and SEMA3B-AS1 were highly expressed in the low risk group (Fig. 2d). Therefore, these studies identified 12 BCSC-related lncRNAs with prognostic significance for breast cancer.

**Table 2:** The risk model of 12 BCSC-related lncRNAs with prognostic value in breast cancer

LncRNA	Coef	HR	HR.95L	HR.95H	p-value	Risk
AL122010.1	− 0.488472	0.61356332	0.40085805	0.9391353	0.02450628	Low
AC245297.3	− 0.39251	0.6753596	0.51409558	0.88720971	0.00480743	Low
Z68871.1	0.675654	1.96531726	1.25113209	3.08718155	0.00336423	High
LINC00578	0.189555	1.20871114	1.02961181	1.41896451	0.0205253	High
LINC01871	− 0.397982	0.67167386	0.51597499	0.87435591	0.00309782	Low
AC097639.1	0.207522	1.23062483	0.94799722	1.59751257	0.11903949	High
AP000851.2	− 0.271595	0.7621631	0.59823533	0.97101016	0.02794552	Low
AP003119.3	0.190492	1.20984515	0.95504527	1.53262399	0.11439212	High
AP001207.3	0.274701	1.31613764	1.12443772	1.54051956	0.00062571	High
LINC00668	0.26132	1.29864313	1.08803894	1.55001251	0.00379653	High
AC022509.2	− 0.211675	0.80922784	0.64043779	1.02250321	0.07614398	Low
SEMA3B-AS1	− 0.466346	0.62729049	0.50223943	0.78347763	3.94E−05	Low

*coef* the coefficient of lncRNAs correlated with survival, *HR* hazard ratio, *HR.95L* low 95%CI of HR, *HR.95H* high 95%CI of HR

### Evaluation of the risk model of the 12 BCSC-related lncRNAs as independent prognostic factor for breast cancer patients

**Fig. 3:**
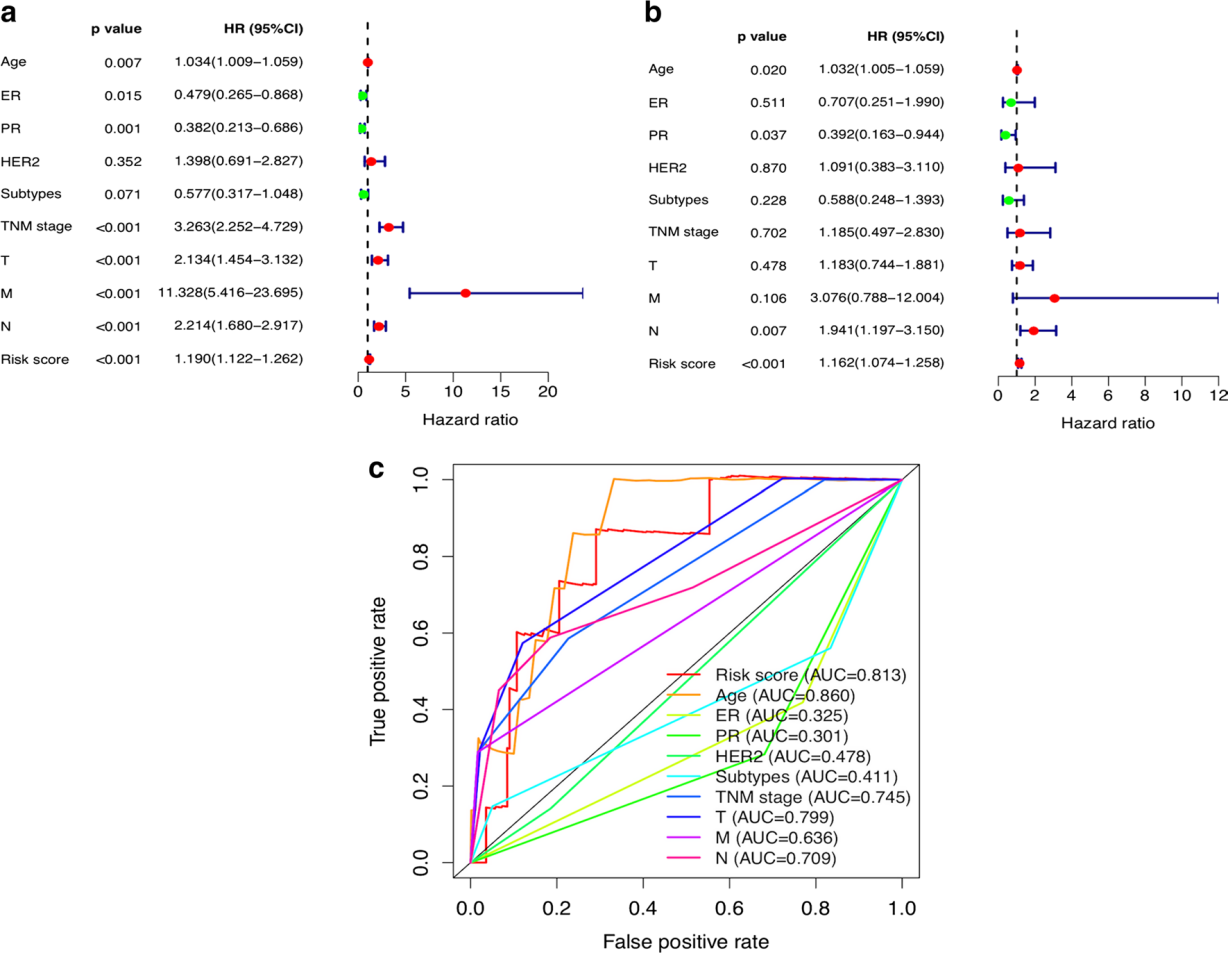
Assessment of the prognostic risk model of the 12 BCSC-related lncRNAs in breast cancer. **a** The univariate and **b** multivariate Cox regression analysis of risk model score and clinical features regarding prognostic value. **c** The AUC for risk model score and clinical features according to the ROC curves. Clinical features: Age, ER, PR, HER2, Subtypes (molecular subtypes), TNM stage, T (tumor size), N (lymph node metastasis) and M (distant metastasis)

Univariate and multivariate Cox regression analyses were performed to assess whether the risk model of the above 12 BCSC-related lncRNAs was an independent prognostic factor for breast cancer. The HR of the risk score and 95% CI were 1.190 and 1.122–1.262 (p < 0.001) in univariate Cox regression analysis (Fig. 3a) and 1.162 and 1.074–1.258 (p < 0.001) in multivariate Cox regression analysis (Fig. 3b), respectively, indicating that the risk model of the 12 BCSC-related lncRNAs was the most significant prognostic factor for breast cancer, independent of clinicopathological parameters such as age, ER expression, PR expression, HER2 expression, molecular subtypes, TNM stage, tumor size, lymph node metastasis and distant metastasis. To evaluate the predictive specificity and sensitivity of the risk score on the prognosis of breast cancer patients, the area under the ROC curve (AUC) of the risk score was estimated. The AUC of the risk score was 0.813, followed by the AUC of age and more than the AUCs of other clinicopathological factors (Fig. 3c), suggesting that the prognostic risk model of the 12 BCSC-related lncRNAs for breast cancer was considerably reliable. All of the above results indicated that the 12 BCSC-related lncRNAs were significant independent prognostic factors for breast cancer patients.

### Correlation of the expression of the 12 BCSC-related lncRNAs with clinicopathological factors

**Fig. 4:**
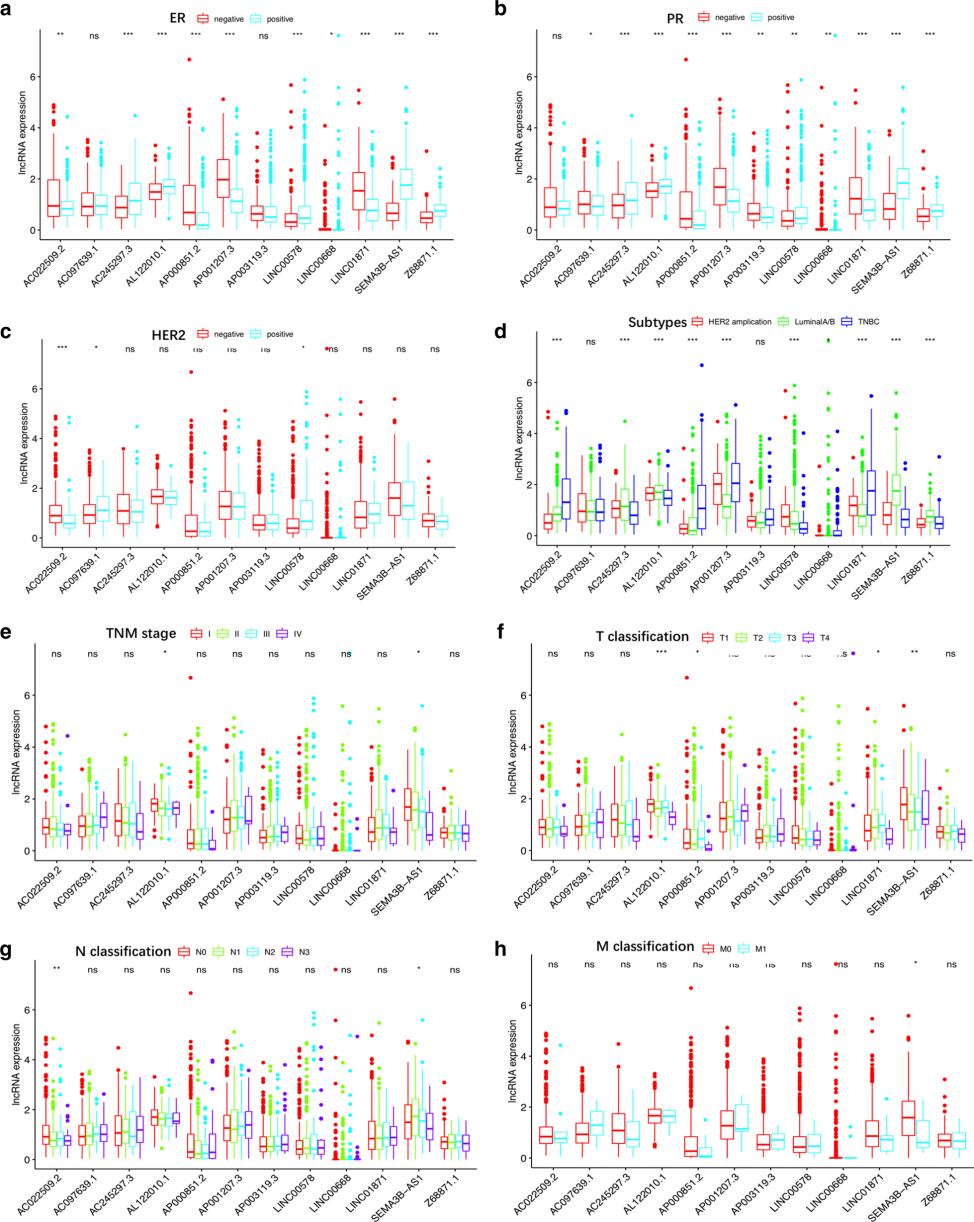
The correlation of the expression of the 12 BCSC-related lncRNAs with clinicopathological factors. **a** ER expression. **b** PR expression. **c** HER2 expression. **d** molecular subtypes (LuminalA/B; HER2 amplification; TNBC: triple-negative breast cancer). **e** TNM stage. **f** Tumor size (T1: < 2 cm; T2: ≥ 2 cm and < 5 cm; T3: ≥ 5 cm; T4: invasion of chest wall and/or skin). **g** N classification (N0: no lymph node metastasis; N1: 1–3 lymph node metastasis; N2: 4–9 lymph node metastasis; N3: ≥ 10 lymph node metastasis). **h** M classification (M0: no distant metastasis; M1: distant metastasis). ns: no statistical significance, *p < 0.05, **p < 0.01, ***p < 0.001, ****p < 0.0001

To further investigate whether the 12 BCSC-related lncRNAs were involved in the development of breast cancer, we assessed the association of the expression of the 12 BCSC-related lncRNAs with clinicopathological factors. There were significant correlations between most of the 12 BCSC-related lncRNAs and ER expression, PR expression and molecular subtypes, as shown in Fig. 4.

### Different stemness statuses in the low-risk and high-risk groups

**Fig. 5:**
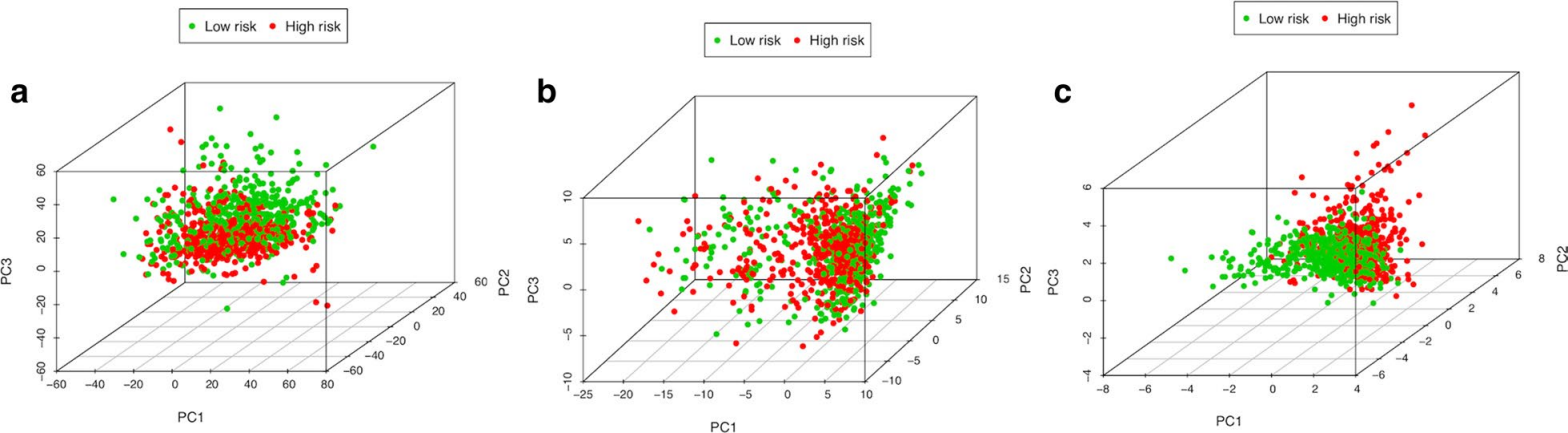
The Low-risk and high-risk groups displayed different stemness statuses. **a**–**c** Principal components analysis (PCA) between the low-risk and high-risk groups based on the whole-genome, BCSC-related encoding genes and the risk model of the 12 BCSC-related lncRNAs expression profiles

**Fig. 6:**
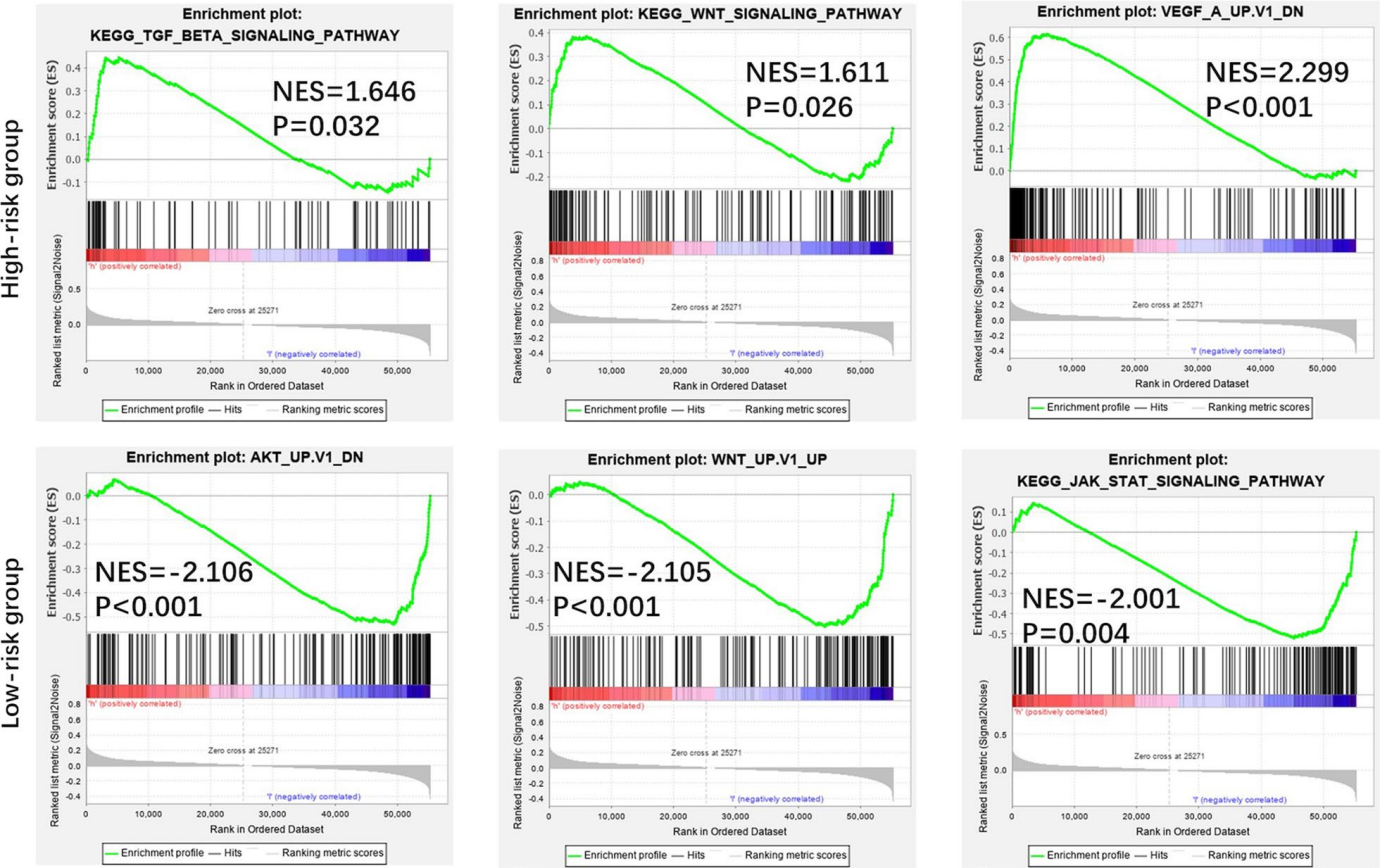
Functional enrichment analysis based on the risk model of the 12 BCSC-related lncRNAs by GSEA. Significantly enriched KEGG pathways and oncogenic signatures in the high-risk and low-risk groups

PCA was performed to compare the difference between the low-risk and high-risk groups based on the risk model of the 12 BCSC-related lncRNAs, 224 BCSC-related encoding genes and whole-genome expression profiles, respectively (Fig. 5). The results showed that the low-risk and high-risk groups based on the risk model were distributed in distinct directions, more obvious than the others, suggesting that the risk model could divide breast cancer patients into two parts and that the stemness status of breast cancer patients in the high-risk group differed from those in the low-risk group. Functional annotation was further conducted using GSEA, and the results showed that the differentially expressed genes between the high-risk and low-risk groups based on the risk model of the 12 BCSC-related genes were enriched in stemness-related processes and CSC-related pathways (Fig. 6). These results indicated that the low-risk and high-risk groups showed different stemness statuses.

## Discussion

In clinical practice, although the OS of breast cancer patients has made promising improvements, the occurrence of drug resistance to breast cancer has constantly increased. The increased frequency of chemoresistance and endocrine resistance might result from enrichment with BCSCs. Because large-scale current therapeutic strategies focus on eliminating the majority of non-CSCs, the residual population of chemo-resistant cancer cells that contributes to relapse and metastasis is thought to result from the existence of minimal residual CSCs [15, 16]. Thus, effective treatment aimed at BCSCs has promising prospects. This led us to find potential specific prognostic biomarkers and therapeutic targets for BCSCs. Recently, an increasing number of studies have suggested the crucial role of lncRNAs in regulating the stemness of CSCs and predicting prognosis in numerous cancers such as intestinal cancer, lung cancer, and hepatocellular cancer [17–19]. Similarly, an increasing number of lncRNAs have been implicated in the regulation of stemness in breast cancer. LncRNA-Hh strengthened CSC generation in twist-positive breast cancer by activating the Hedgehog signaling pathway [20]. LncRNA FEZF1-AS1 promoted the stemness of BCSC tumorigenesis by targeting the miR-30a/Nanog axis [21]. LncRNA HOTAIR contributed to the epithelial–mesenchymal transition (EMT) of BCSCs by activating the STAT3 signaling pathway [22]. In this study, we identified the risk model of the 12 BCSC-related lncRNAs as an independent prognostic factor for breast cancer. To date, among these 12 BCSC-related lncRNAs, only LINC00578, LINC00668 and SEMA3B-AS1 have been studied in breast cancer or other cancers. It has been reported that LINC00578 is associated with worse OS in pancreatic cancer and lung adenocarcinoma [23, 24]. LINC00668 promoted tumorigenesis and progression and indicated poor prognosis in not only breast cancer but also other cancers, such as colorectal cancer, hepatocellular carcinoma and non-small-cell lung cancer [25–28]. SEMA3B-AS1 might serve as a new tumor suppressor to inhibit the development of hepatocellular carcinoma, esophageal squamous cell carcinoma and gastric cardia adenocarcinoma [29–31]. Consistent with our results, LINC00578 and LINC00668, as high-risk BCSC-related lncRNAs, were correlated with worse prognosis in breast cancer patients, whereas SEMA3B-AS1, as a low-risk BCSC-related lncRNA, was associated with better prognosis in breast cancer patients. Breast cancer is a complex disease and highly heterogeneous tumor [32]. To assess survival prognosis and guide individual therapeutic decisions, breast cancer has been divided into distinct molecular subtypes based primarily on the expression status of hormonal receptors such as ER, PR, HER2 and Ki67 (tumor proliferation index) as follows: luminal A/B (ER and/or PR positive), HER2 enriched (HER2 positive) and triple-negative breast cancer (ER, PR and HER2 negative) [33]. It is also well known that ER and/or PR positive indicates an effective endocrine therapy outcome and better survival prognosis; HER2 positive represents a more aggressive phenotype but is sensitive to HER2-targeted therapy; triple negative is enriched with BCSCs and associated with a worse prognosis due to the lack of effective therapeutic targets [34, 35]. Moreover, it was demonstrated that distinct molecular subtypes of breast cancer were enriched with different amounts of BCSCs [4]. Thus, distinct ER, PR and HER2 statuses indicated different biological processes of breast cancer and survival outcomes. In line with the abovementioned findings, most of the 12 BCSC-related lncRNAs were remarkably associated with ER expression, PR expression and molecular subtypes, which further suggested that the 12 BCSC-related lncRNAs might be involved in the development and progression of breast cancer, and the risk model was also based on the intrinsic properties of breast cancer. More interestingly, there were no significant relationship between most of the 12 BCSCs-related lncRNAs and tumor size (T), lymph node (N) status, distant metastasis (M) and TNM stage, indicating that the risk model of the 12 BCSCs-related lncRNAs has no close correlation with the sooner or later for finding and diagnosis of breast cancer, but is only strongly linked to the intrinsic biological characteristics of distinct subtypes of breast cancer. Furthermore, the risk model was the second most statistically significant prognostic signature compared with other clinicopathological factors, and the ROC result (AUC = 0.813) confirmed that the risk model is reliable. Combined with the AUC of the risk model score, these results all indicated that the risk model of the 12 BCSC-related lncRNAs had superior prognostic value to other clinicopathological factors. The results of PCA and GSEA functional annotation illustrated that the high-risk and low- risk groups showed different distribution directions and aggregation centers based on the risk model, rather than the whole-genome expression profiles and BCSC-related genes expression profiles, indicating that the significant differences in OS between the high-risk and low-risk groups might result from different stemness and oncogenic statuses induced by the risk model of the 12 BCSC-related lncRNAs. Taken together, these results indicated that the prognostic signature of the 12 BCSC-related lncRNAs might be a feasible independent prognostic factor for breast cancer in clinical practice. To date, a key challenge of precision genomic medicine is to make reliable and accurate predictions of clinical outcomes from high-dimensional molecular data [36]. To solve this problem, there have been some advances in Cox regression with prognostic value in recent years. A Cox elastic net has been used in objective and data-driven feature selection with time-to-event data [37]. Cox-nnet is an artificial neural network approach that has been utilized in predicting low-dimensional survival prognosis [38]. Bayesian-optimized deep survival models (SurvivalNet models) have successfully improved the accuracy of prognostic prediction for high-dimensional cancer genomic profiles [39]. In addition, Cox-nnet has a better performance than SurvivalNet models, and SurvivalNet models provide comparable performance to the Cox elastic net [39, 40]. Moreover, Cox-PASNet, which is a novel pathway-based sparse deep neural network for survival analysis that integrates high-dimensional genomic data and clinical data, has been applied to identify significant prognostic factors [40]. However, our study has some limitations. We applied traditional univariate and multivariate Cox proportional hazards analyses to establish and estimate the prognostic value of the risk model of the 12 BCSC-related lncRNAs. Although the method has been approved and employed in many researches, it is necessary to improve our further study with more advanced methodologies and technologies in the future. To further validate our bioinformatics prediction results, in-depth studies on the 12 BCSC-related lncRNAs, including functional experiments and molecular mechanisms, are needed.

## Conclusion

In conclusion, we identified a BCSC-related lncRNA signature consisting of 12 lncRNAs (Z68871.1, LINC00578, AC097639.1, AP003119.3, AP001207.3, LINC00668, AL122010.1, AC245297.3, LINC01871, AP000851.2, AC022509.2 and SEMA3B-AS1), which can act as a novel independent prognostic factor for breast cancer. In the future, with prospective validation, the 12 BCSC-related lncRNA signature may improve the predictive accuracy and guide individual specific therapy for patients with breast cancer.

## Supplementary information

**Additional file 1.** The list of 1198 BCSC-related lncRNAs.

**Additional file 2.** The list of 213 BCSC-related mRNAs.

**Additional file 3: Fig. S1.** Protein-protein interaction network of the 213 BCSC-related encoding genes (mRNAs).

## Data Availability

All data utilized in this study are included in this article and all data supporting the findings of this study are available on reasonable request from the corresponding author.

## Abbreviations

LncRNAs: Long noncoding RNAs
TCGA: The Cancer Genome Atlas
ROC: Receiver operating characteristics
PCA: Principal components analysis
GSEA: Gene Set Enrichment Analysis
OS: Overall survival
HR: Hazard ratio
ER: Estrogen receptor
PR: Progesterone receptor
HER2: Human epidermal growth factor receptor 2
