# Supplementary figures and images for: Identification and validation of stemness-related lncRNA prognostic signature for breast cancer
Source: J Transl Med. 2020 Aug 31;18:331. doi: 10.1186/s12967-020-02497-4 (PMC7461324; doi:10.1186/s12967-020-02497-4)

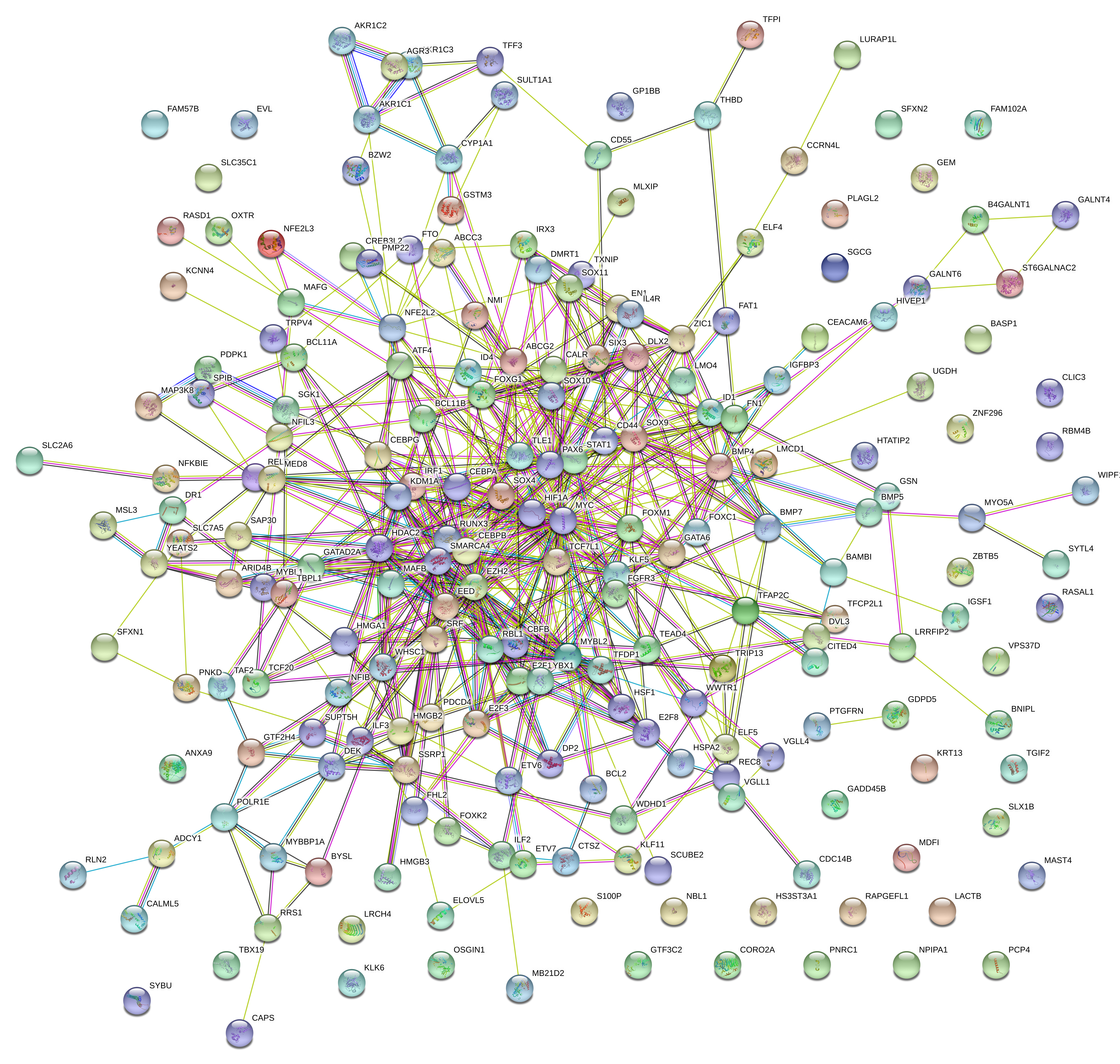

Supplement: Supplementary file 3 — Additional file 3: Fig. S1. Protein-protein interaction network of the 213 BCSC-related encoding genes (mRNAs). [file 12967_2020_2497_MOESM3_ESM.png]
